# Harvesting more grain zinc of wheat for human health

**DOI:** 10.1038/s41598-017-07484-2

**Published:** 2017-08-01

**Authors:** Xin-Ping Chen, Yue-Qiang Zhang, Yi-Ping Tong, Yan-Fang Xue, Dun-Yi Liu, Wei Zhang, Yan Deng, Qing-Feng Meng, Shan-Chao Yue, Peng Yan, Zhen-Ling Cui, Xiao-Jun Shi, Shi-Wei Guo, Yi-Xiang Sun, You-Liang Ye, Zhao-Hui Wang, Liang-Liang Jia, Wen-Qi Ma, Ming-Rong He, Xi-Ying Zhang, Chang-Lin Kou, Yan-Ting Li, De-Shui Tan, Ismail Cakmak, Fu-Suo Zhang, Chun-Qin Zou

**Affiliations:** 1 College of Resources and Environmental Sciences, China Agricultural University, Beijing, 100193 China; 2 College of Resources and Environment, Southwest University, Chongqing, 400716 China; 3 Institute of Genetics and Developmental Biology, Chinese Academy of Sciences, Beijing, 100101 China; 4 College of Resources and Environmental Sciences, Nanjing Agricultural University, Nanjing, 210095 China; 5 Soil and Fertilizer Research Institute, Anhui Academy of Agricultural Sciences, Hefei, 230031 China; 6 College of Resources and Environmental Sciences, Henan Agricultural University, Zhengzhou, 450000 China; 7 Northwest Agriculture & Forestry University, Yangling, 712100 China; 8 Institute of Agro-resources and Environment, Hebei Academy of Agricultural & Forestry Sciences, Shijiazhuang, 050051 China; 9 College of Resources and Environmental Sciences, Agricultural University of Hebei, Baoding, 071001 China; 10 College of Agronomy, Shandong Agricultural University, Tai’an, 271000 China; 11 The Center for Agricultural Resources Research, IGDB, CAS, Hebei, 050021 China; 12 Henan Academy of Agricultural Sciences, Zhengzhou, 450000 China; 13 Institute of Agricultural Resource and Regional Planning, Chinese Academy of Agricultural Sciences, Beijing, 100081 China; 14 Institute of Agricultural Resources and Environment, Shandong Academy of Agricultural Sciences, Jinan, 250100 China; 15 Faculty of Engineering and Natural Sciences, Sabanci University, 34956 Istanbul, Turkey

## Abstract

Increasing grain zinc (Zn) concentration of cereals for minimizing Zn malnutrition in two billion people represents an important global humanitarian challenge. Grain Zn in field-grown wheat at the global scale ranges from 20.4 to 30.5 mg kg^−1^, showing a solid gap to the biofortification target for human health (40 mg kg^−1^). Through a group of field experiments, we found that the low grain Zn was not closely linked to historical replacements of varieties during the Green Revolution, but greatly aggravated by phosphorus (P) overuse or insufficient nitrogen (N) application. We also conducted a total of 320-pair plots field experiments and found an average increase of 10.5 mg kg^−1^ by foliar Zn application. We conclude that an integrated strategy, including not only Zn-responsive genotypes, but of a similar importance, Zn application and field N and P management, are required to harvest more grain Zn and meanwhile ensure better yield in wheat-dominant areas.

## Introduction

Zinc is a very important micronutrient for human health. However, human Zn deficiency (ZnD), which affects about two billion people worldwide (mostly pregnant women and children under five years old), is a widespread global health burden especially in developing countries^1^. Regions with human ZnD are mostly where soil Zn deficiency occurs, indicating their inherent connection^1, 2^. It’s also known that the most common cause of human ZnD is chronic inadequate dietary intake of Zn, particularly in areas with high cereal and low animal products consumption^3^. Unfortunately, the potential of agriculture system to supply nutritious food to overcome “hidden hunger” (e.g., micronutrient malnutrition) for human health has received less attention than the common malnutrition and related issues (such as calorie intake, food demand, crop yield and environmental sustainability)^4–6^. However, micronutrient deficiency including ZnD is a more widespread problem than low energy intake in many parts of the world, and probably will be more serious in the future because of climate change^7^. A crucial challenge for agriculture is therefore to realize a more nutritious agricultural production system while simultaneously ensuring food, resource and environment security under changing global climate^8^.

In last two decades, developing micronutrient-enriched staple food crops to alleviate human micronutrient deficiencies (biofortification) has increasingly been considered in addition to traditional strategies such as supplementation, food fortification and dietary diversification^9^. Cereal-based foods represent the largest proportion of the daily micronutrient intake in countries with high incidence of micronutrient malnutrition^4^. Biofortified staple foods by either genetic (e.g., plant breeding) or agronomic strategies can increase micronutrient intake for the resource-poor people who consume them daily, and thus complement existing strategies^3^.

Wheat is a major source of dietary calories, protein and minerals for the world’s growing population^10^. In recent decades, the “Green Revolution” significantly increased wheat yield globally and therefore reduced common malnutrition remarkably^11^. Nowadays, sufficient Zn in wheat grain is expected to meet part of Zn requirement for human health in many countries^3, 9, 12^. Nutritionists suggested that Zn in wheat grain should reach a target of around 40 mg kg^−1^ to avoid human ZnD, a micronutrient malnutrition risk for human health^3, 4^.

However, today the grain Zn status of field-grown wheat at large scale is still unclear^10, 13^. Furthermore, there is a lack of integrative evaluation on changes of grain Zn in wheat as affected by measures of the “Green Revolution” including variety changes and fertilizaiton^11, 14^. While, strategies are urgent to mitigate or even biofortify the declining grain Zn in wheat under increasing negative conditions^7, 8^. Therefore, we focus on three questions: (i) How large is the gap between current wheat grain Zn and the target for human health in global and regional scales? (ii) Do the measures used to improve yield in the “Green Revolution” affect grain Zn in wheat? (iii) Can a strategy be built up for harvesting more grain Zn and grain yield at same time? To address these questions, we conducted multi-site/year field experiments and also used meta-analysis of published data.

## Results and Discussion

### The gap between current wheat grain Zn and the target

#### Meta-analysis on global scale

**Figure 1:**
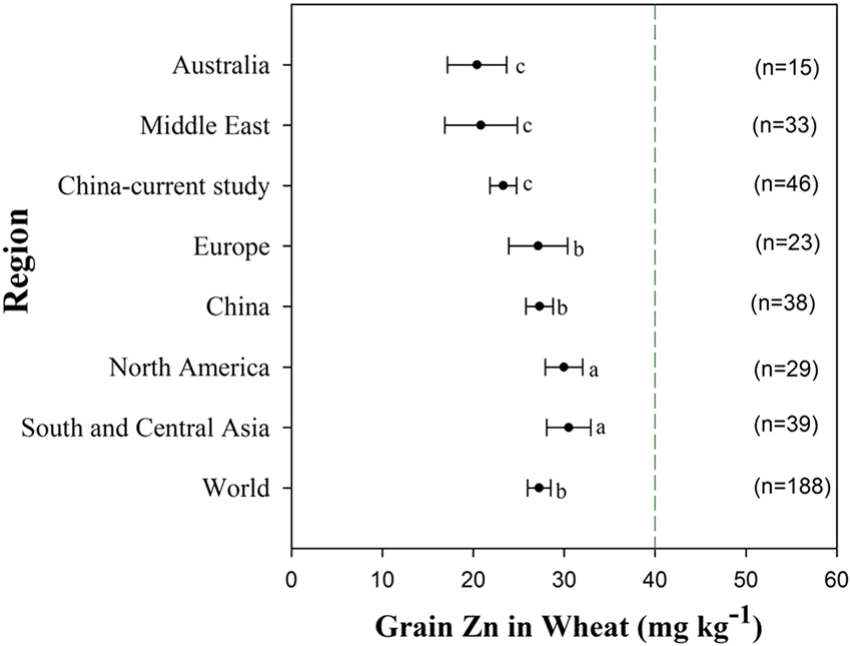
The gap between current wheat grain Zn and the target in global scale. Details of published data source are listed in Supplementary Table 1. The black dots indicate the averaged values and the error bars represent 95% confidence intervals. Different letters on columns indicate significant differences among medians of columns at *P* < 0.05 level by nonparametric test of independent samples via SPSS statistics. The green dash line indicates the target Zn concentration (40 mg kg^−1^) for wheat biofortification^3, 4^. The observation number of each set was shown in parenthesis.

By a meta-analysis through published data from 109 available studies (Supplementary Table 1), we found that grain Zn in wheat grown in major wheat producing regions worldwide ranges from 20.4 to 30.5 mg kg^−1^ with a grand mean of 27.3 mg kg^−1^ (Fig. 1). These variations in grain Zn represent interacting consequences of multiple factors including cultivars, soils and environments^12^. However, these concentrations are much lower than the target concentration (around 40 mg kg^−1^) set by nutritionists for biofortification^3, 4^, with a gap of 10 to 20 mg kg^−1^. On regional scales, grain Zn in wheat is much lower in Middle East where diets based on wheat predominate and human ZnD is widespread^1, 12^. Consumption of unleavened bread made by wheat flour further decreases Zn availability to population in this region, because the phytate could not be digested and Zn cannot be released during the processing of unleavened bread^15^. In addition, India and Pakistan have been suggested as top priorities for wheat biofortification with Zn, due to the prevalence of human ZnD, huge wheat planting areas, and a population relying on wheat based diets^13^. We found that the reported grain Zn in wheat grown in South Asia is indeed lower than the target concentration, although it is higher than in other regions (Fig. 1).

#### Measurement in China

China is the largest wheat producer and consumer worldwide, producing 18.0% of the world harvest in 2013 (http://faostat.fao.org/) and half of Chinese population relies on wheat-based food especially in North China. To understand the grain Zn status of wheat harvested in China, we conducted a total of 46 site-year of on-farm field experiments at 26 sites in seven provinces within the major agro-ecological areas of wheat production in China during 2008 to 2012, and a total of 955 grain samples were collected from plots managed with current farmers’ practices^16^. The results show that grain Zn in wheat ranges from 10.1 to 49.7 mg kg^−1^ with an average of 23.3 mg kg^−1^ (Table 1), comparable to our literature survey (Fig. 1). Current Zn status in China averages higher than that in Australia and the Middle East, and is similar or lower than that in Europe, North America and South and Middle Asia (Fig. 1). Today, 100 million people are estimated to suffer from ZnD among Chinese rural poor, and it has been ranked as one of the top-five priority countries for biofortification intervention at the national level^13^. Consequently, biofortifying wheat with Zn would be meaningful in China and could be a paradigm for the world.

**Table 1:** Grain Zn concentration of wheat grown in major wheat cropping systems in China.

Cropping system	Grain Zn concentration in wheat (mg kg^−1^)					
	Sample number	Min	Max	Median^*^	Mean	CV%
Wheat in semi-arid/arid area (10%)	76	11.7	31.0	17.9c	18.6	21.0
Wheat rotated with maize (65%)	759	10.1	49.7	22.4b	23.4	26.1
Wheat rotated with rice (20%)	120	18.0	41.5	24.3a	25.5	20.2
Total	955	10.1	49.7	22.4	23.3	26.0

The numbers in parenthesis represent yield proportion of each cropping system to total wheat production in China.

*Medians with the different letters indicate significantly different at *P* < 0.05 level by nonparametric test of independent samples via SPSS statistics.

As shown in Table 1 and Supplementary Fig. 1, bread wheat is mainly grown in three major cropping systems in China: (*i*) wheat in semi-arid/arid areas (W-S); (*ii*) wheat rotated with maize (W-M) in the North China Plain and (*iii*) rotated with rice (W-R) in the Yangzi river. At a subnational scale, grain Zn concentrations in wheat differ significantly among these three cropping systems (Table 1). Furthermore, the dietary structure among people in these three cropping systems differs greatly, as wheat-based staple food makes up more than 70% of total staple food in semi-arid/arid areas and less than 20% in wheat-rice rotation systems (Supplementary Fig. 1). Wheat grown in semi-arid/arid areas is lowest in grain Zn (Table 1), indicating that poor soil conditions (such as high soil pH and low soil available Zn) and the prevalence of drought limits Zn uptake and thereafter translocation to grain^2, 12^. These soil conditions are also typical for low available P to plant roots, and therefore, heavy application of P fertilizers under such low P conditions is a very common practice which may further reduce Zn uptake and accumulation in plants^2^. Therefore, semi-arid/arid area should be given the highest priority for wheat biofortification with Zn - not only in China, but also in many other developing countries, such as in Middle East, South and Central Asia, where people suffer more from Zn deficiency^1^.

### Did the “Green Revolution” technologies cause low grain Zn of wheat?

The “Green revolution” was a remarkable scientific and technological achievement in the 20th century; it was based largely on the intensification of management on land already under agriculture, accomplished through the use of high-yielding crop varieties, chemical fertilizers and pesticides, irrigation, and mechanization^6^. It increased food production substantially and fed billions of people during the last fifty years^11^. On the other hand, there has been debate on whether the “Green Revolution” technologies led to an enlarged prevalence of micronutrient malnutrition in developing countries where these modern cropping systems were adopted because little thought was given to nutritional value and its effects on human health in the processes of cereal breeding and cropping system changes^9, 17^. This concern is more concentrated in wheat than in other cereals. Some studies have shown that wheat grain Zn concentrations are negatively correlated with grain yield or cultivar release year among diverse wheat cultivars and regions^10, 14^. However, management factors (e.g. fertilization) have not been considered carefully in such studies. To address this issue, we have pursued a second group of experiments to explore the response of grain Zn in wheat to major measures of the “Green Revolution” technologies, including cultivar changes and nitrogen (N) and phosphorus (P) fertilization.

#### Cultivars and grain Zn

**Figure 2:**
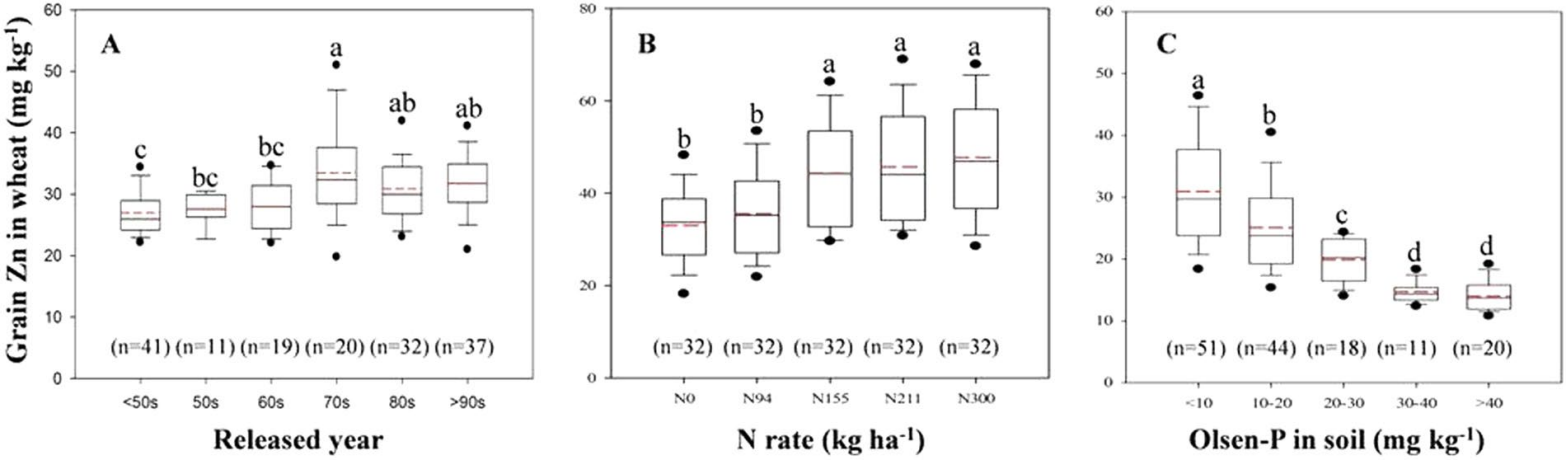
Measures of the “Green Revolution” including historical shift of varieties (**A**), N application rate (**B**) and elevated soil P concentration due to continuous application of P fertilizer (**C**) affect grain Zn in wheat grown in China. These historical varieties were grown in the same field at the North China Plain. And their grain Zn concentration (Fig. 2A), height (Supplementary Fig. 3A), and thousand kernel weight (TKW, Supplementary Fig. 3B) were shown. The variety number of each set was shown in parenthesis. For N rate (Fig. 2B and Supplementary Fig. 5A), N0, N94, N155 (optimal N rate), N211 and N300 (N rate of farmers’ practice) indicates the rates of N application at 0, 94, 155, 211 and 300 kg ha^−1^, respectively. The sample numbers are 32 for each N rate. Elevated soil P concentration (Fig. 2C and Supplementary Fig. 5B) is presented as Olsen-P concentration which is the most used indicator for soil P status. The sample numbers for ranges of Olsen-P in soil were shown in parenthesis. Range of solid and red dashed lines in this figure indicate median and mean, respectively. The box boundaries indicate the 75% and 25% quartiles, the whisker caps indicate 90th and 10th percentiles, and the circles indicate the 95th and 5th percentiles. Medians of columns with the different letters indicate significantly different at *P* < 0.05 level by nonparametric test of independent samples via SPSS statistics.

By planting 160 historically extensive wheat cultivars at the same location in North China Plain, we found that the heights of these cultivars are obviously declined with the released years (Supplementary Fig. 3A), while grain sizes (thousand kernel weight) are substantially rising until 70s in last century after which reaching a fluctuant plateau (Supplementary Fig. 3B). Such trends in Chinese wheat were coherent with global trends that breeding and adoption of dwarfing modern cultivars are one of landmark characteristics of the “Green Revolution”^11^. Furthermore, grain Zn concentrations ranged from 20.6–40.8 mg kg^−1^, with a slight increasing trend with release year (Fig. 2A). Similar trends were also reported in Mexico^8^ and Siberia^18^, although these results are not generally expected^4, 14^. This inconsistency may be explained in part by the fact that the ancient parents used for wheat breeding in China may have genetically low Zn concentrations in grain^19^. For example, variety “Chinese spring” is a famous parent for wheat breeding, and its grain Zn concentration has a very low range from 20 to 30 mg kg^−1^ in different studies^14, 20^. Furthermore, the positive and significant correlation between grain Zn concentration and grain size among these historical wheat varieties indicates that dilution effects of increasing yields on grain Zn concentration may not necessarily occur (Supplementary Fig. 4). This illuminates the possibility of combining high grain Zn with high grain yield in the future advanced cultivars^4, 10^.

#### Nitrogen fertilization and grain Zn

The application of N and/or P fertilizers has become a common agricultural practice for yield increase in most agriculture systems^5, 21^. In a six-year field experiment with bread wheat, it was found that no or low N application (N0 and N94) caused low grain Zn concentration, while an optimal N rate (N155) for grain yield resulted in higher grain Zn concentration (Fig. 2B); however, increasing N rates over the optimal level had no significant effect on grain Zn concentration as well as grain N concentration (Fig. 2B and Supplementary Fig. 5A). This is because N-containing compounds (e.g., Zn-chelators or Zn-transporting proteins) represent critical players in uptake, xylem transport, remobilization via phloem and the size of sink strength for Zn accumulation in wheat grain^22, 23^. In practice, China is undergoing a transformation from overuse of N to reasonable N input in cereal production^16^, and our findings highlight that transformation will have no negative effect on grain Zn concentration in wheat. However, nowadays N deficiency is still a problem in many developing countries, which may not only limit yield improvement, but also be a reason for low Zn concentration in grain.

#### Phosphorus fertilization and grain Zn

Phosphorus applied in excess of crop P demands can results in the build-up of soil P concentrations^21^. We found an increase in grain P but a dramatic decrease in grain Zn of field-grown wheat in a calcareous soil due to elevated soil P concentration (measured as Olsen-P) after continuous P application for four crop seasons (Fig. 2C and Supplementary Fig. 5B). Phosphorus-induced decline in grain Zn of wheat may be due to a combination of several processes, including reduced plant-availability of Zn in the rhizosphere, reduction in Zn uptake per unit of root weight, decreasing mycorrhizal colonization, diminished root-to-shoot translocation of Zn, and yield-induced dilution effect^24, 25^. We also demonstrate a faster decline of grain Zn within the range of soil Olsen-P from 3.4 to 40 mg kg^−1^. On average, Olsen-P concentrations in agricultural soils of China have been increased from 7.4 to 24.7 mg kg^−1^ during the last three decades^26^. Consequently, grain Zn concentrations in wheat may have decreased significantly due to elevated soil P concentration, with the potential for an aggravated prevalence of Zn malnutrition in areas with intensive soil P accumulation such as China, India, Pakistan and some countries in the Middle East^21^ where Zn deficiency in soil, crop and population is widespread^1, 2^. In addition, increasing root P uptake in P-enriched soils also increases grain phytate concentration^24, 25^ that has depressive effects on Zn bioavailability in diet^12^. Thus, to overcome human Zn deficiency by either genetic or agronomic biofortification will require optimal P management^21, 26^ with attention to P-Zn antagonism in the soil-crop-human continuum^25^.

We conclude that adoption of new cultivars in the Green Revolution has not necessarily decreased grain Zn in wheat, while overuse of P or inefficient N use has played a more dominant role in decreasing Zn accumulation in wheat grain. Nutrient imbalance in agricultural systems vary substantially among regions and countries with different economic development, for instance phosphorus overuse is very popular in some “fast-developing” counties, and insufficient nitrogen application is still a problem in some “slow-developing” countries^21, 27^; the findings presented suggest that we can increase wheat yield and simultaneously harvest more Zn in wheat grain through optimal N and P management.

### Integrated strategies for Zinc biofortification

#### Foliar Zn application

**Figure 3:**
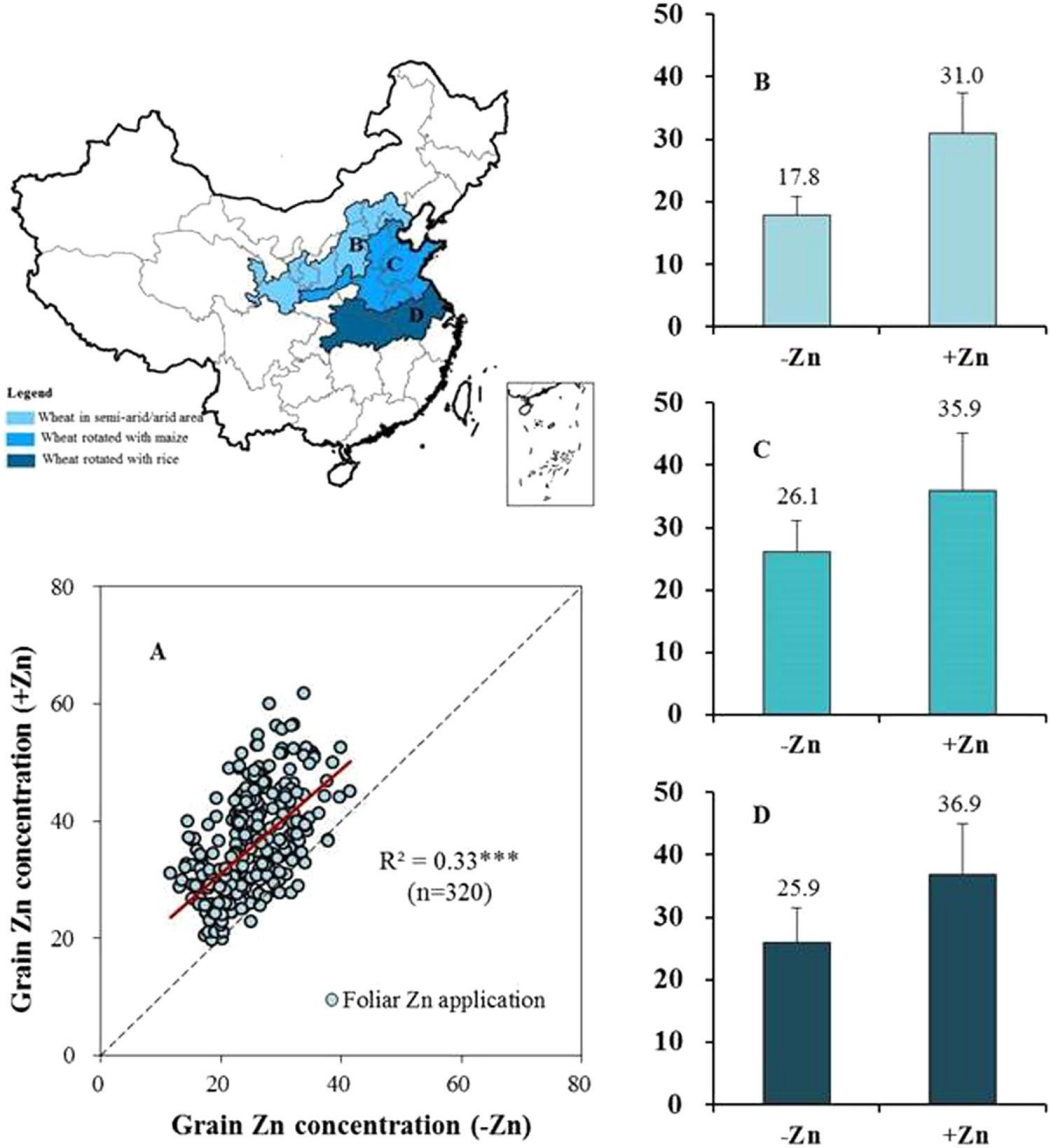
Foliar Zn application affects grain Zn (mg kg^−1^) in wheat grown in three wheat cropping systems of China. (**A**) Linear regression between grain Zn concentration without and with foliar Zn application cross all locations from three cropping system. (**B**) Cropping system of wheat in semi-arid/arid area (W-S). (**C**) Cropping system of wheat rotated with maize (W-M). (**D**) Cropping system of wheat rotated with rice (W-R). Sample numbers for (**B**,**C** and **D**) were 48, 216 and 56, respectively. Figures 3A–D was generated by Microsoft Excel (version 2010, Microsoft Corporation, USA). The map at county scale of China (www.gadm.org) was generated by using ArcGis software (desktop vision 9.3, Esri, USA, www.esri.org). They were combined with Supplementary Fig. 1 by using Adobe Photoshop software (version 13.0.0, Adobe Systems, USA, www.adobe.com).

Beside N and P management, to test the effects of another agronomic approach-Zn application on Zn biofortification of wheat, we conducted a third group of experiments including a total of 320-pair plots in seven provinces in China during 2008 to 2012 (Fig. 3), in which applied supplementary Zn to foliage. Compared with no Zn application, foliar application of Zn fertilizers (+Zn) increased grain Zn concentrations from 24.9 to 35.4 mg kg^−1^ on average. A linear regression (red line) across all plots showed an average increase of 10.5 mg kg^−1^ by foliar Zn application (Fig. 3A). By cropping system studied, increases were 13.2, 9.8 and 11.0 mg kg^−1^ in the W-S, W-M, and W-R systems, causing increases of 74%, 37% and 43%, respectively (Fig. 3B–D). These findings suggest that foliar-applied Zn is important to maintain a sufficient pool of physiologically available Zn within plant tissues for re-translocation of Zn to grain after flowering^28^. The current effectiveness of foliar Zn application (>10 mg kg^−1^ in average) has a measurable biological impact on human health. The increases in grain Zn by foliar Zn spray occurs in grain fractions such as in bran and endosperm where the increased Zn has high bioavailability^28^. Furthermore, foliar Zn application involves no yield tradeoff^29^; in fact, there is more or less yield increase by foliar Zn application according to previous or current studies (Supplementary Table 2). Without doubt, foliar application of Zn fertilizers represents an effective strategy to biofortify wheat with Zn.

#### Genetic biofortification

Genetic biofortification is a strategy that uses plant breeding techniques to produce new varieties of staple food crops with higher micronutrient levels and higher amounts of substances that promote bioavailability and intestinal absorption of micronutrients^3^. Plant breeding strategy represents a sustainable and cost-effective solution to malnutrition problems by exploring natural genetic variation to develop mineral-dense crop varieties^9, 30^. Literature review showed substantial genetic variation in grain Zn concentrations among wheat germplasm resources that can be exploited in breeding programs (Supplementary Table 3). However, field tests using the Zn-biofortified lines together with check varieties have just started in recent years under the HarvestZinc project (www.harvestzinc.org). Comparing available studies, elite lines in multiple field trials showed an increase of grain Zn by 6.5 to 10 mg kg^−1^ compared to local checks (Supplementary Table 3), indicating another pathway to biofortify wheat with Zn in order to overcome human ZnD. Although great progresses in breeding and releasing Zn-enriched wheat varieties have been made^3, 10^, this approach also faces uncertainties such as yield tradeoff or unstable traits of grain Zn under varied soils and environments^12, 30^.

#### Integrated strategy to harvest more grain zinc

**Figure 4:**
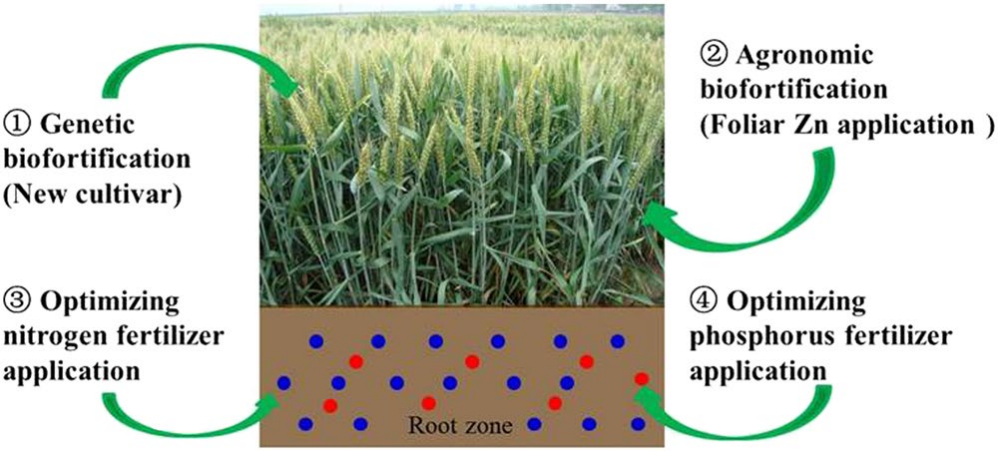
Conceptual framework for an integrated strategy to harvest more grain zinc while ensuring high yield and protecting the environment.

For developing countries, economic development and thereby improving people’s welfare would be the ultimate solution to break the malnutrition-poverty trap and finally overcome malnutrition including human ZnD by affording and accessing animal source foods^30^. Specific to developing countries in regions with wheat-based diets, integrative strategies are still needed to overcome Zn malnutrition in the short and middle terms, because ongoing trends in agriculture and climate can depress grain Zn concentration in wheat due to negative climatic and soil factors including increasing atmospheric CO_2_ concentration^7^, overuse of P or inefficient N use^21, 27^, depleting the already low available Zn in soil^12^, and drought prevalence in many wheat growing regions^31^. No single intervention strategy may work effectively to deal with all these negative impacts. Therefore, we propose a conceptual framework for an integrated strategy to harvest more grain zinc while ensuring high yields and protecting the environment (Fig. 4). Under this framework, at least four factors should be managed in coordination in a sustainable and intensified agriculture system: (*i*) adoption of biofortified cultivars with characteristics of Zn-enriched grain, high yield and high resistance to stresses; (*ii*) building up a sufficient pool of available Zn in wheat shoots for Zn retranslocation to grain after flowering by efficient and economical foliar application of Zn; (*iii*) optimizing application rate of N fertilizer that ensures better yield and grain Zn concentration with lower environmental cost; and (*iv*) avoiding overuse of P fertilizer and keeping soil available P at appropriate levels that meet yield requirements and minimize P-Zn antagonism interactions and environmental pollution. Under such a scenario, combining agronomic and genetic strategies will harvest more wheat grain Zn in way that will rapidly meet the target for biofortification.

## Methods

### Meta-analysis of grain Zn concentrations in field-grown wheat at global scale

Meta-analysis of available published data referred to grain Zn concentration in field-grown wheat was conducted through “Web of Science” (www.webofknowledge.com). The used keywords in this databank are “wheat” plus “grain” plus “zinc”. The publishing period of these literatures is before December 2015 when Meta-analysis begins. Those potential literatures are further checked whether they were conducted in the field conditions. Finally, items including region, country, numbers of data point (DP, defines as the product of cultivar, site and crop season), observations (defines as the averaged value of grain Zn concentration in field-grown bread wheat without Zn application at a site), source of publications were recorded (Supplementary Table 1). Thus, regional comparison can be realized by ranging these data by regions which represent 80% of global wheat production^32^. Considering the great variation of numbers of data point in various studies, un-weighted mean (observations) of different regions were used to compare directly in Fig. 1. Meanwhile, weighted mean $(=\frac{{\rm{\Sigma }}({\rm{Observation}}\times {\rm{DP}})}{{\rm{\Sigma }}\mathrm{DP}})$ of different regions were also calculated for reference (Supplementary Fig. 2).

### Field survey of grain Zn in wheat grown in major wheat cropping systems in China

Geographic distribution of major wheat cropping system in China is shown in Supplementary Fig. 1. Wheat grown in semi-arid/arid areas (W-S), wheat-maize rotation (W-M), and wheat-rice rotation (W-R) are the three major systems of wheat cropping in China, and each contributes about 10%, 65% and 20% of total wheat product, respectively^33^. In addition, wheat made up 76.8, 72.5 and 15.1% of staple cereal consumption in regions with W-S, W-M and W-R crop system respectively, according to China National Nutrient and Health Survey^34^. During 2008 to 2012, a total of 46 site-years of field experiments (6, 33, and 7 in W-S, W-M, W-R and systems, respectively) were conducted within the major agro-ecological areas of wheat production in China (Supplementary Fig. 1). Each field experiment includes a treatment that followed farmers’ practices in the region but was conducted in experimental plots^16^. At maturity, in total of 955 grain samples were collected from plots with current practice in each experiment and then their Zn concentrations were determined using ICP-AES after digestion by H_2_O_2_-HNO_3_^29^.

### Grain Zn concentrations of historically popularized wheat cultivars in China

160 winter wheat varieties, mainly cultivated in North China Plain (NCP) where is the main wheat production area in China including all of W-M system and a part of W-S system (Supplementary Fig. 1), were grown in a field with the original plan to reproduce seeds during 2006–2007 cropping season on the experimental station of Institute of Genetics and Developmental Biology, Chinese Academy of Science, Beijing. Here we used to explore the relationship between grain Zn concentrations of these varieties and their release years.

### Field experiments with different rates of N or P fertilizer

During year 2007 to 2013, field experiment with wheat treated by varied N rates was continuously conducted at Quzhou County, Hebei Province which locates in the North China Plain. Five N rates with each four replications were applied in these field plots (300 m^2^ plot^−1^) by a randomized complete block experimental design. These N rates were listed as follows: no N application (N0), half or less than half of optimal N application (94 kg ha^−1^ in average, N94), optimal N application (155 kg ha^−1^ in average, N155), one and a half times of optimal N application (211 kg ha^−1^ in average, N211), and farmers’ N application (300 kg ha^−1^, N300). Optimal N rate was based on root zone nutrient management and may vary in each season^35^.

During year 2009 to 2012, field experiment with wheat treated by varied P rates was also conducted at Quzhou County. Six P rates with each four replicates were applied in split plots (43.2 m^2^ plot^−1^) by a randomized complete block experimental design. These P rates with calcium superphosphate were 0, 25, 50, 100, 200 and 400 kg ha^−1^, respectively.

More information about these two experiments could be referred to previous studies^16, 25, 36^. At maturity, grains were sampled in both experiments for measuring grain Zn concentrations. In addition, soil samples from latter experiment were also collected for measuring soil available P (Olsen-P) due to its residual effect.

### Field experiments with foliar Zn application

We design series of field experiment with two treatments (one without Zn application, the other with foliar Zn application) to test the effectiveness of foliar Zn application on increasing grain Zn concentration of wheat at major wheat cropping systems in China. The foliar Zn application treatment consisted of two times of foliar Zn application at the heading and milk stages. At each time of foliar Zn application, 0.4% (w/v) of aqueous solution of ZnSO_4_·7H_2_O with 800 litres per hectare were used at the very late afternoon until most of the leaves were wet^29^. During 2008 to 2012, we have finished a total of 320-pair plots (48, 216, and 56 in W-S, W-M and W-R systems, respectively). At maturity, grains were sampled in each plot and then used to determine grain Zn concentrations universally at China Agricultural University by ICP-AES method^29^.

## Electronic supplementary material

Supplementary Information
