# Supplementary material for: Harvesting more grain zinc of wheat for human health
Source: Sci Rep. 2017 Aug 1;7:7016. doi: 10.1038/s41598-017-07484-2 (PMC5539200; doi:10.1038/s41598-017-07484-2)
Supplement: Supplementary file 1 — Supplementary Information [file 41598_2017_7484_MOESM1_ESM.doc]

**Harvesting more grain zinc of wheat for human health**

Xin-Ping Chen^a,1^, Yue-Qiang Zhang^b,a,1^, Yi-Ping Tong^c^, Yan-Fang Xue^a^, Dun-Yi Liu^a^, Wei Zhang^a^, Yan Deng^a^, Qing-Feng Meng^a^, Shan-Chao Yue^a^, Peng Yan^a^, Zhen-Ling Cui^a^, Xiao-Jun Shi^b^, Shi-Wei Guo^d^, Yi-Xiang Sun^e^, You-Liang Ye^f^, Zhao-Hui Wang^g^, Liang-Liang Jia^h^, Wen-Qi Ma^i^, Ming-Rong He^j^, Xi-Ying Zhang^k^, Chang-Lin Kou^l^, Yan-Ting Li^m^, De-Shui Tan^n^, Ismail Cakmak^o^, Fu-Suo Zhang^a^ and Chun-Qin Zou^a,*^

**Supplementary Information**


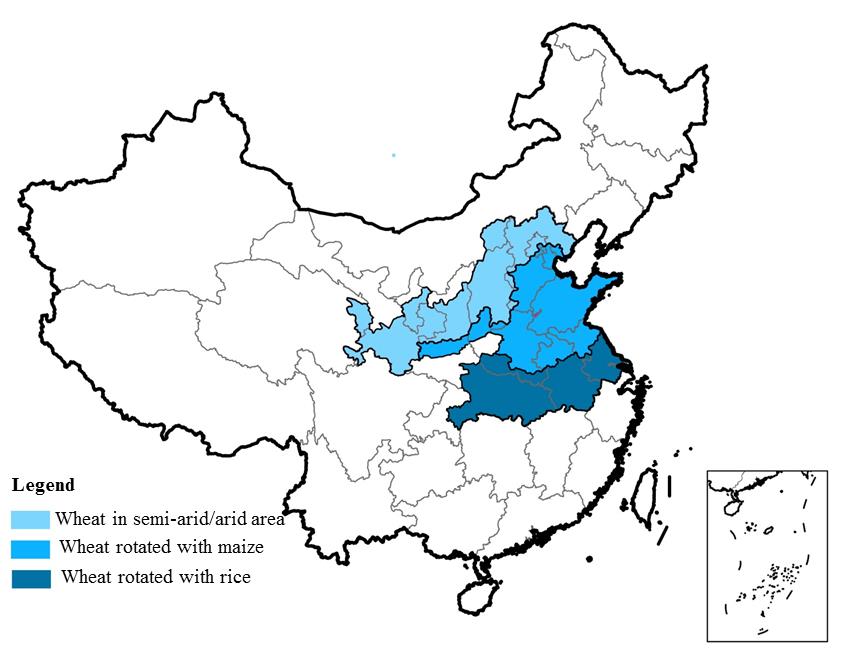


**Supplementary Fig. 1. Geographic distribution of major wheat cropping system in China**. Wheat-maize rotation (W-M), wheat-rice rotation (W-R) and wheat grown in semi-arid/arid areas (W-S) are the three major systems of wheat cropping in China, and each contributes about 65%, 20% and 10% of total wheat product, respectively. In addition, wheat made up 76.8, 72.5 and 15.1% of staple cereal consumption in regions with W-S, W-M and W-R crop system respectively, according to China National Nutrient and Health Survey in 2002. This figure was generated by the following steps: (1) get information about wheat total yield and wheat cropping system at county level of China from National Bureau of Statistics of China; (2) and then separate these counties with different wheat cropping systems on open-accessed base map at county scale of China (www.gadm.org) by using ArcGis software (desktop vision 9.3, Esri, USA, www.esri.org).

**Supplementary Fig. 2. The gap between current wheat grain Zn and the target in global scale (weighted result)**. Details of published data source are listed in Supplementary Table 1. The numbers of data points (DP) of each set were showed in parenthesis. The green dash line indicates the target Zn concentration (40 mg kg^-1^) for wheat biofortification. Because there is only one value for a given region under weighted condition, no error bar could be shown. While error bars for 95% confidence intervals under un-weighted condition are shown only for integrity.

**Supplementary Fig. 3.** **Height (A) and thousand kernel weight (TKW, B) of historical shift of wheat varieties grown in China.** These historical varieties were grown in the same field at the North China Plain. The variety number of each set was showed in parenthesis. Range of solid and red dashed lines in this figure indicate median and mean, respectively. The box boundaries indicate the 75% and 25% quartiles, the whisker caps indicate 90th and 10th percentiles, and the circles indicate the 95th and 5th percentiles. Medians of columns with the different letters indicate significantly different at *P*<0.05 level by nonparametric test of independent samples via SPSS statistics.


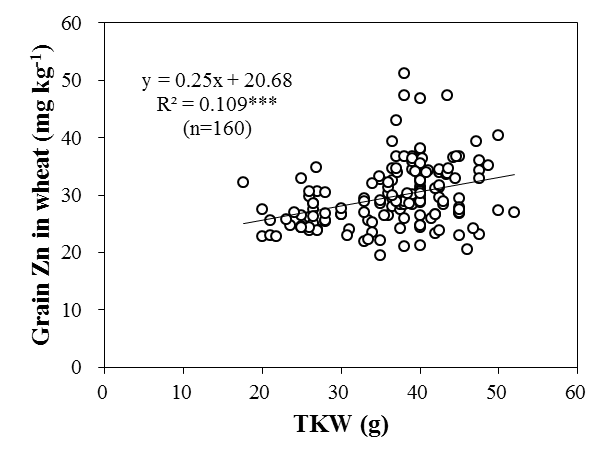


**Supplementary Fig. 4.** **Correlation between thousand kernel weight (TKW) and grain Zn concentration of historical wheat varieties in China.** These historical varieties were grown in the same field at the North China Plain. ***, indicates significant correlation at *P*<0.01 level by regression analysis via SPSS statistics. The variety number was showed in parenthesis.

**Supplementary Fig. 5. Nitrogen application rate (A) and elevated soil P concentration (measured as Olsen-P) due to continuous application of P fertilizer (B) increase grain N or P in wheat grown in China.** For N rate, N0, N94, N155 (optimal N rate), N211 and N300 (N rate of farmers’ practice) indicates the rates of N application at 0, 94, 155, 211 and 300 kg ha^-1^, respectively. The sample numbers are 32 for each N rate. For elevated soil P concentration, the sample numbers are 51, 44, 18, 11 and 20 for ranges of Olsen-P in soil. Range of solid and red dashed lines in this figure indicate median and mean, respectively. The box boundaries indicate the 75% and 25% quartiles, the whisker caps indicate 90th and 10th percentiles, and the circles indicate the 95th and 5th percentiles. Medians of columns with the different letters indicate significantly different at *P*<0.05 level by nonparametric test of independent samples via SPSS statistics.

**Supplementary Table 1. Appendix of data source in Figure 1.** Available published references about grain Zn concentration in field-grown wheat without Zn application, including 109 references with 188 observations of mean value at each site.

| **Region** | **Country** | **Data point^*^** | **Grain Zn concentration (mg kg^-1^)** | | | **Reference** |
| --- | --- | --- | --- | --- | --- | --- |
|  |  |  | **Mean (observation)** | **Min** | **Max** |  |
| Australia | Australia | 8 | 9.8 | - | - | 1 |
| Australia | Australia | 42 | 25.2 | 19.9 | 30.9 | 2 |
| Australia | Australia | 28 | 16.3 | 12.4 | 20.1 | 2 |
| Australia | Australia | 8 | 12.2 | 10.9 | 14.1 | 3 |
| Australia | Australia | 2 | 20.2 | 19.9 | 20.5 | 3 |
| Australia | Australia | 4 | 21.5 | - | - | 4 |
| Australia | Australia | 4 | 15.8 | - | - | 4 |
| Australia | Australia | 3 | 21.1 | - | - | 5 |
| Australia | Australia | 2 | 27.5 | - | - | 6 |
| Australia | Australia | 78 | 28.0 | 14.0 | 46.0 | 7 |
| Australia | Australia | 20 | 19.2 | 7.0 | 35.4 | 8 |
| Australia | Australia | 21 | 22.0 | 16.0 | 35.0 | 9 |
| Australia | New Zealand | 1 | 29.8 | - | - | 10 |
| Australia | New Zealand | 1 | 14.8 | - | - | 10 |
| Australia | New Zealand | 1 | 23.0 | - | - | 10 |
| Africa | Zambia | 1 | 24.3 | - | - | 28 |
| East Asia | China | 1 | 31.8 | - | - | 11 |
| East Asia | China | 1 | 26.6 | - | - | 12 |
| East Asia | China | 1 | 25.3 | - | - | 12 |
| East Asia | China | 1 | 35.0 | - | - | 12 |
| East Asia | China | 1 | 20.8 | - | - | 12 |
| East Asia | China | 1 | 24.8 | - | - | 12 |
| East Asia | China | 19 | 27.6 | 21.4 | 33.5 | 13 |
| East Asia | China | 23 | 25.0 | 19.3 | 37.9 | 13 |
| East Asia | China | 1 | 24.3 | - | - | 14 |
| East Asia | China | 8 | 28.7 | 26.0 | 30.5 | 15 |
| East Asia | China | 76 | 22.8 | 17.7 | 30.9 | 16 |
| East Asia | China | 48 | 26.2 | 20.7 | 31.2 | 16 |
| East Asia | China | 62 | 23.5 | 16.2 | 31.2 | 16 |
| East Asia | China | 1 | 34.4 | - | - | 17 |
| East Asia | China | 1 | 25.9 | - | - | 17 |
| East Asia | China | 1 | 26.5 | 16.0 | 28.5 | 18 |
| East Asia | China | 3 | 29.1 | - | - | 19 |
| East Asia | China | 265 | 32.3 | 21.4 | 58.2 | 20 |
| East Asia | China | 43 | 28.6 | 21.2 | 34.8 | 21 |
| East Asia | China | 10 | 24.1 | - | - | 22 |
| East Asia | China | 10 | 31.6 | - | - | 22 |
| East Asia | China | 10 | 19.4 | - | - | 22 |
| East Asia | China | 40 | 27.8 | 12.0 | 80.0 | 23 |
| East Asia | China | 1 | 27.3 | - | - | 24 |
| East Asia | China | 4 | 30.7 | - | - | 25 |
| East Asia | China | 28 | 26.8 | 21.0 | 38.1 | 25 |
| East Asia | China | 32 | 29.4 | 20.0 | 44.1 | 26 |
| East Asia | China | 5 | 28.9 | - | - | 27 |
| East Asia | China | 2 | 28.6 | - | - | 28 |
| East Asia | China | 2 | 19.2 | - | - | 28 |
| East Asia | China | 69 | 30.4 | - | - | 29 |
| East Asia | China | 586 | 30.3 | - | - | 29 |
| East Asia | China | 126 | 41.8 | 20.9 | 108.9 | 30 |
| East Asia | China | 2 | 21.0 | - | - | 31 |
| East Asia | China | 240 | 29.3 | 19.9 | 43.9 | 32 |
| East Asia | China | 3 | 24.2 | 23.9 | 24.4 | 33 |
| East Asia | China | 2 | 22.3 | 21.9 | 22.7 | 34 |
| East Asia | China | 4 | 25.1 | 23.0 | 29.3 | 35 |
| East Asia | Japan | 9 | 47.4 | 20.5 | 93.6 | 36 |
| Europe | Croatia | 12 | 35.1 | 30.5 | 42.5 | 37 |
| Europe | Croatia | 12 | 30.7 | 23.5 | 41.2 | 37 |
| Europe | Croatia | 40 | 18.2 | 14.9 | 22.9 | 38 |
| Europe | France | 153 | 20.5 | 16.1 | 27.2 | 39 |
| Europe | France | 18 | 21.0 | 16.0 | 28.0 | 39 |
| Europe | France | 33 | 24.7 | 20.0 | 33.0 | 39 |
| Europe | France | 175 | 25.0 | - | - | 39 |
| Europe | Hungary | 150 | 21.4 | 13.5 | 34.5 | 40 |
| Europe | Poland | 2 | 21.6 | - | - | 41 |
| Europe | Poland | 3 | 50.0 | - | - | 42 |
| Europe | Poland | 3 | 32.5 | - | - | 43 |
| Europe | Russia | 24 | 34.2 | - | - | 44 |
| Europe | Serbia | 14 | 33.2 | 26.6 | 44.3 | 45 |
| Europe | Serbia | 36 | 21.2 | - | - | 46 |
| Europe | Sweden | 6 | 28.2 | 25.0 | 54.0 | 47 |
| Europe | Sweden | 3 | 30.5 | - | - | 48 |
| Europe | Sweden | 120 | 27.4 | - | - | 49 |
| Europe | Sweden | 606 | 27.0 | - | - | 49 |
| Europe | Sweden | 5 | 25.0 | - | - | 49 |
| Europe | UK | 4 | 21.1 | 18.9 | 23.5 | 50 |
| Europe | UK | 6 | 18.4 | 11.6 | 29.3 | 8 |
| Europe | UK | 5 | 21.4 | 19.6 | 24.0 | 51 |
| Europe | UK | 24 | 36.2 | - | - | 52 |
| Middle East | Egypt | 18 | 42.0 | - | - | 53 |
| Middle East | Egypt | 2 | 22.0 | - | - | 54 |
| Middle East | Egypt | 4 | 43.7 | 40.7 | 46.7 | 55 |
| Middle East | Iran | 1 | 22.0 | - | - | 56 |
| Middle East | Iran | 10 | 44.0 | 36.0 | 57.0 | 57 |
| Middle East | Iran | 137 | 31.6 | 11.7 | 64.0 | 58 |
| Middle East | Iran | 5 | 13.9 | - | - | 59 |
| Middle East | Iran | 5 | 14.3 | - | - | 59 |
| Middle East | Iran | 1 | 31.2 | - | - | 60 |
| Middle East | Iran | 1 | 31.2 | - | - | 60 |
| Middle East | Iran | 4 | 32.8 | - | - | 58 |
| Middle East | Iran | 60 | 9.9 | - | - | 61 |
| Middle East | Iran | 60 | 18.6 | - | - | 61 |
| Middle East | Iran | 4 | 13.5 | - | - | 62 |
| Middle East | Iran | 20 | 19.0 | - | - | 63 |
| Middle East | Iran | 100 | 30.7 | 12.8 | 48.7 | 64 |
| Middle East | Israel | 16 | 27.0 | 15.0 | 61.0 | 65 |
| Middle East | Turkey | 2 | 10.5 | - | - | 66 |
| Middle East | Turkey | 1 | 34.5 | - | - | 67 |
| Middle East | Turkey | 1 | 11.1 | - | - | 67 |
| Middle East | Turkey | 1 | 26.0 | - | - | 67 |
| Middle East | Turkey | 1 | 6.1 | - | - | 68 |
| Middle East | Turkey | 1 | 8.4 | - | - | 68 |
| Middle East | Turkey | 1 | 7.9 | - | - | 68 |
| Middle East | Turkey | 1 | 13.2 | - | - | 68 |
| Middle East | Turkey | 30 | 7.0 | - | - | 69 |
| Middle East | Turkey | 30 | 9.0 | - | - | 69 |
| Middle East | Turkey | 4 | 10.0 | - | - | 70 |
| Middle East | Turkey | 2 | 9.8 | - | - | 71 |
| Middle East | Turkey | 8 | 26.0 | 23.0 | 28.0 | 65 |
| Middle East | Turkey | 2 | 25.8 | - | - | 28 |
| Middle East | Turkey | 2 | 12.9 | - | - | 28 |
| Middle East | Turkey | 64 | 22.5 | 10.4 | 38.2 | 72 |
| North America | Canada | 10 | 32.2 | 22.4 | 45.8 | 73 |
| North America | Canada | 15 | 35.0 | - | - | 74 |
| North America | Canada | 15 | 30.2 | - | - | 74 |
| North America | Canada | 15 | 30.5 | - | - | 74 |
| North America | Canada | 15 | 28.5 | - | - | 74 |
| North America | Canada | 15 | 24.6 | - | - | 74 |
| North America | Canada | 15 | 30.2 | - | - | 74 |
| North America | Canada | 605 | 32.8 | - | - | 75 |
| North America | Canada | 591 | 32.7 | - | - | 75 |
| North America | Canada | 408 | 34.9 | - | - | 75 |
| North America | Canada | 6 | 37.4 | 29.5 | 44.8 | 76 |
| North America | Canada | 4 | 29.1 | 26.1 | 32.8 | 8 |
| North America | Canada | 8 | 20.0 | 16.0 | 25.2 | 77 |
| North America | Mexico | 33 | 30.4 | 16.9 | 60.8 | 78 |
| North America | Mexico | 132 | 35.0 | 25.2 | 53.3 | 79 |
| North America | Mexico | 40 | 26.8 | - | - | 80 |
| North America | Mexico | 40 | 34.0 | - | - | 80 |
| North America | Mexico | 1 | 21.0 | - | - | 28 |
| North America | USA | 8 | 38.0 | 33.0 | 48.0 | 81 |
| North America | USA | 1 | 36.0 | 31.0 | 45.0 | 81 |
| North America | USA | 112 | 33.8 | 23.0 | 43.0 | 82 |
| North America | USA | 14 | 27.0 | 24.0 | 29.0 | 82 |
| North America | USA | 6 | 20.9 | - | - | 83 |
| North America | USA | 250 | 30.5 | 14.9 | 43.3 | 84 |
| North America | USA | 162 | 31.5 | 19.5 | 53.3 | 85 |
| North America | USA | 14 | 20.9 | 16.0 | 26.3 | 86 |
| North America | USA | 14 | 29.3 | 26.1 | 33.9 | 86 |
| North America | USA | 40 | 36.3 | 17.0 | 70.5 | 87 |
| North America | USA | 26 | 20.0 | 16.0 | 35.2 | 8 |
| South America | Argentina | 1 | 37.7 | - | - | 88 |
| South America | Argentina | 1 | 36.9 | - | - | 88 |
| South America | Argentina | 1 | 37.2 | - | - | 89 |
| South America | Argentina | 1 | 52.5 | - | - | 89 |
| South America | Argentina | 80 | 26.7 | - | - | 90 |
| South America | Argentina | 34 | 29.2 | 7.8 | 56.4 | 91 |
| South America | Brazil | 1 | 30.8 | - | - | 92 |
| South America | Brazil | 1 | 26.3 | - | - | 92 |
| South America | Brazil | 20 | 59 | 40 | 85 | 93 |
| South and Central Asia | Central Asia | 123 | 29.0 | 22.0 | 39.0 | 94 |
| South and Central Asia | Central Asia | 100 | 28.0 | 23.0 | 33.0 | 94 |
| South and Central Asia | India | 12 | 37.4 | 23.8 | 66.7 | 95 |
| South and Central Asia | India | 4 | 51.5 | 42.3 | 55.6 | 96 |
| South and Central Asia | India | 2 | 38.7 | - | - | 97 |
| South and Central Asia | India | 2 | 39.5 | - | - | 97 |
| South and Central Asia | India | 1 | 9.6 | - | - | 98 |
| South and Central Asia | India | 1 | 25.6 | - | - | 98 |
| South and Central Asia | India | 2 | 24.1 | - | - | 99 |
| South and Central Asia | India | 2 | 24.7 | - | - | 100 |
| South and Central Asia | India | 40 | 28.5 | - | - | 80 |
| South and Central Asia | India | 40 | 32.1 | - | - | 80 |
| South and Central Asia | India | 40 | 31.3 | - | - | 80 |
| South and Central Asia | India | 40 | 32.3 | - | - | 80 |
| South and Central Asia | India | 40 | 44.1 | - | - | 80 |
| South and Central Asia | India | 1 | 38.4 | - | - | 101 |
| South and Central Asia | India | 2 | 31.1 | - | - | 102 |
| South and Central Asia | India | 1 | 29.0 | - | - | 28 |
| South and Central Asia | India | 2 | 40.2 | - | - | 28 |
| South and Central Asia | India | 2 | 26.4 | - | - | 28 |
| South and Central Asia | India | 2 | 24.4 | - | - | 103 |
| South and Central Asia | India | 100 | 37.0 | 26.6 | 47.1 | 104 |
| South and Central Asia | Kazakhstan | 504 | 28.0 | 23.0 | 33.0 | 94 |
| South and Central Asia | Kazakhstan | 2 | 21.5 | - | - | 28 |
| South and Central Asia | Pakistan | 11 | 32.2 | 24.0 | 36.0 | 105 |
| South and Central Asia | Pakistan | 9 | 29.8 | 24.0 | 36.0 | 105 |
| South and Central Asia | Pakistan | 9 | 27.3 | 24.0 | 36.0 | 105 |
| South and Central Asia | Pakistan | 11 | 27.3 | 24.0 | 36.0 | 105 |
| South and Central Asia | Pakistan | 3 | 27.8 | - | - | 106 |
| South and Central Asia | Pakistan | 65 | 28.8 | 23.7 | 36.0 | 107 |
| South and Central Asia | Pakistan | 40 | 34.2 | - | - | 80 |
| South and Central Asia | Pakistan | 40 | 29.3 | - | - | 80 |
| South and Central Asia | Pakistan | 10 | 27.0 | - | - | 108 |
| South and Central Asia | Pakistan | 1 | 27.0 | - | - | 28 |
| South and Central Asia | Pakistan | 1 | 29.0 | - | - | 28 |
| South and Central Asia | Pakistan | 2 | 42.9 | - | - | 28 |
| South and Central Asia | Pakistan | 2 | 32.7 | - | - | 28 |
| South and Central Asia | Pakistan | 60 | 20.4 | 16.0 | 23.0 | 109 |
| South and Central Asia | Pakistan | 60 | 21.7 | 18.0 | 25.0 | 109 |

^*^ Data point defines as the product of cultivar, site and crop season.

**Supplementary Table 2. The summary of wheat yield response to foliar Zn fertilizer treatment from previous studies and current study.**

| Country for field trials | Yield response to foliar Zn application (%) | Reference |
| --- | --- | --- |
| China | 0.0 | 28 |
| China | 2.8 | 112 |
| China | 4.9 | 113 |
| China | 1.6 | Current study |
| India | 0.0 | 28 |
| India | 0.8 | 114 |
| Kazakhstan | 1.8 | 28 |
| Mexico | 3.8 | 28 |
| Pakistan | 10.9 | 28 |
| Turkey | 2.8 | 28 |

**Supplementary Table 3. Breeding achievement of biofortified varieties with zinc in field trials.**

| Country for field trials | Yield level (t ha^-1^) | Grain Zn concentration (mg kg^-1^) | | | | Increase (mg kg^-1^) | | | Reference |
| --- | --- | --- | --- | --- | --- | --- | --- | --- | --- |
|  |  | Local checks | Biofortified varieties | | | Min | Max | Mean |  |
|  |  |  | Min | Max | Mean |  |  |  |  |
| India | 3.0-5.0 | - | - | - | - | 5.0 | 10.0 | 8.5 | 110 |
| Pakistan | 3.0-5.0 | - | - | - | - | 7.0 | 14.0 | 10.0 | 110 |
| India, | 3.0-5.0 | 32.8 | 29.0 | 39.5 | 32.5 | 0.0 | 8.0 | 0.0 | 80 |
| Pakistan and Mexico | - | 21.7 | 14.1 | 53.0 | 28.2 | 0.0 | 23.8 | 6.5 | 111 |

**Supplementary Table 4. Complete data source list in Supplementary Table 1, Supplementary Table 2, and Supplementary Table 3**.

| **Reference number** | **Reference (Author list, title, journal, volume and pages, year published)** |
| --- | --- |
|
| 1 | Graham, R. D. *et al*. Selecting zinc-efficient cereal genotypes for soils of low zinc status. *Plant Soil* **146,** 241-250 (1992). |
| 2 | McDonald, G. K. *et al*. A simple method to evaluate genetic variation in grain zinc concentration by correcting for differences in grain yield. *Plant Soil* **306,** 49-55 (2008). |
| 3 | Peck, A. W. *et al.* Zinc nutrition influences the protein composition of flour in bread wheat (*Triticum aestivum* L.). *J. Cereal Sci.* **47,** 266-274 (2008). |
| 4 | Ryan, M. H. *et al*. Grain mineral concentrations and yield of wheat grown under organic and conventional management. *J. Sci. Food Agr.* **84,** 207-216 (2004). |
| 5 | Zubaidi, A. *et al.* Nutrient uptake and distribution by bread and durum wheat under drought conditions in South Australia. *Aust. J. Exp. Agr.* **39,** 721-732 (1999). |
| 6 | Fernando, N. *et al.* Rising atmospheric CO_2_ concentration affects mineral nutrient and protein concentration of wheat grain. *Food Chem.* **133,** 1307-1311 (2012). |
| 7 | Murphy, G. M. ＆ Law, D. P. Some mineral levels in Australian wheat. *Aust. J. Exp. Agric. Animal Hus.* **14,** 663-665 (1974). |
| 8 | Batten, G. Concentrations of elements in wheat grains grown in Australia, North America, and the United Kingdom. *Aust. J. Exp. Agr.* **34,** 51-56 (1994). |
| 9 | White, C. L., Robson, A. D. ＆ Fisher, H. M. Variation in nitrogen, sulfur, selenium, cobalt, manganese, copper and zinc contents of grain from wheat and 2 lupin species grown in a range of Mediterranean environments. *Aust. J. Exp. Agr.* **32,** 47-59 (1981). |
| 10 | Curtin, D., Martin, R. J. ＆ Scott, C. L. Wheat (*Triticum aestivum*) response to micronutrients (Mn, Cu, Zn, B) in Canterbury, New Zealand. *New Zeal. J. Crop Hort. Sci.* **36,** 169-181 (2008). |
| 11 | Yang, X. *et al*. Impacts of phosphorus and zinc levels on phosphorus and zinc nutrition and phytic acid concentration in wheat (*Triticum aestivum* L.). *J. Sci. Food Agr.* **91,** 2322-2328 (2011). |
| 12 | Zhang, Y. Q. *et al*. Zinc biofortification of wheat through fertilizer applications in different locations of China. *Field Crops Res.* **125,** 1-7 (2012). |
| 13 | Hou, N., Li, B. ＆ Tong, Y. Study on the effects of nitrogen deficiency on grain iron and zinc concentrations of landrace and bred wheat varieties in China. *J. Plant Nutr.* **34,** 830-838 (2011). |
| 14 | Karim, M. R. *et al*. Alleviation of drought stress in winter wheat by late foliar application of zinc, boron, and manganese. *J. Plant Nutr. Soil Sci.* **175,** 142-151(2012). |
| 15 | Dang, H. *et al*. Absorption, accumulation and distribution of zinc in highly-yielding winter wheat. *Agr. Sci. China* **9,** 965-973 (2010). |
| 16 | Schroeder, J. I. *et al*. Using membrane transporters to improve crops for sustainable food production. *Nature* **497,** 60-66 (2013). |
| 17 | Shi, R. L. *et al*. Identification of quantitative trait locus of zinc and phosphorus density in wheat (*Triticum aestivum* L.) grain. *Plant Soil* **306,** 95-104 (2008). |
| 18 | Shi, R. L. *et al*. Influence of long-term nitrogen fertilization on micronutrient density in grain of winter wheat (*Triticum aestivum* L.). *J. Cereal Sci.* **51,** 165-170 (2010). |
| 19 | Zhang, Y. Q. *et al*. Iron and zinc concentrations in grain and flour of winter wheat as affected by foliar application. *J. Agr. Food Chem.* **58,** 12268-12274 (2010). |
| 20 | Zhang, Y. Q. *et al.* Mineral element concentrations in grains of Chinese wheat cultivars. *Euphytica* **174,** 303-313 (2010). |
| 21 | Tang, J. *et al*. Mineral element distributions in milling fractions of Chinese wheats. *J. Cereal Sci.* **48,** 821-828 (2008). |
| 22 | Zhao, H. *et al.* Effects of wheat origin, genotype, and their interaction on multielement fingerprints for geographical traceability. *J. Agr. Food Chem.* **60,** 10957-10962 (2012). |
| 23 | Huang, M. *et al.* Heavy metals in wheat grain: Assessment of potential health risk for inhabitants in Kunshan, China. *Sci. Total Environ.* **405,** 54-61(2008). |
| 24 | Zhang, Y. *et al.* Effect of source-sink manipulation on accumulation of micronutrients and protein in wheat grains. *J. Plant Nutr. Soil Sci.* **175,** 622-629 (2012). |
| 25 | Lu, X. C. *et al.* Effect of Zn supplementation on Zn concentration of wheat grain and Zn fractions in potentially Zn-deficient soil. *Cereal Res. Commun.* **40,** 385-395 (2012). |
| 26 | Zhao, H. *et al.* Multi-element composition of wheat grain and provenance soil and their potentialities as fingerprints of geographical origin. *J. Cereal Sci.* **57,** 391-397 (2013). |
| 27 | Feng, J. *et al.* Spatial distribution and controlling factors of heavy metals contents in paddy soil and crop grains of rice-wheat cropping system along highway in East China. *Enviro. Geochem. Hlth.* **34,** 605-614 (2012). |
| 28 | Zou, C. *et al*. Biofortification of wheat with zinc through zinc fertilization in seven countries. *Plant Soil* **361,** 119-130 (2012). |
| 29 | Liu, H. *et al*. Grain iron and zinc concentrations of wheat and their relationships to yield in major wheat production areas in China. *Field Crops Res.* **156,** 151-160 (2014). |
| 30 | Wang, C. *et al.* The influences of soil properties on Cu and Zn availability in soil and their transfer to wheat (*Triticum aestivum* L.) in the Yangtze River delta region, China. *Geoderma* **193,** 131-139 (2013). |
| 31 | Wang, J. *et al.* Different increases in maize and wheat grain zinc concentrations caused by soil and foliar applications of zinc in Loess Plateau, China. *Field Crops Res.* **135,** 89-96 (2012). |
| 32 | Zhang, Y. *et al.* Variation of major mineral elements concentration and their relationships in grain of Chinese wheat. *Scientia Agricultura Sinica* **40,** 1871-1876 (2007). |
| 33 | Zhang, S. L. *et al.* Long-term effects of straw and manure on crop micronutrient nutrition under a wheat-maize cropping system. *J*. *Plant Nutr*. **38,** 742-753 (2015). |
| 34 | Li, M. *et al.* Zn distribution and bioavailability in whole grain and grain fractions of winter wheat as affected by applications of soil N and foliar Zn combined with N or P. *J*. *Cereal Sci*. **61,** 26-32 (2015). |
| 35 | Wang, S. *et al.* Foliar zinc, nitrogen, and phosphorus application effect on micronutrient concentrations in winter wheat. *Agron*. *J*. **107,** 61-70 (2015). |
| 36 | Wang, Y. *et al.* Stable isotope labelling and zinc distribution in grains studied by laser ablation ICP-MS in an ear culture system reveals zinc transport barriers during grain filling in wheat. *New Phytol*. **189,** 428-437 (2011). |
| 37 | Sabo, M. ＆ Ugarcic-Hardi, Z. Concentration of macro- and microelements in grain of some new winter wheat genotypes (*triticum aestivum*L.). *Acta Alimentaria* **31,** 235-242 (2002). |
| 38 | Ivezic, V. *et al.* Prediction of trace metal concentrations (Cd, Cu, Fe, Mn and Zn) in wheat grain from unpolluted agricultural soils. *Acta Agr. Scand. B-S. P*. **63,** 360-369 (2013). |
| 39 | Oury, F. X. *et al.* Genetic variability and stability of grain magnesium, zinc and iron concentrations in bread wheat. *Eur*. *J*. *Agron*. **25,** 177-185 (2006). |
| 40 | Asare-Marfo, D. *et al*. Prioritizing countries for biofortification interventions using country-level data. HarvestPlus Working Paper. 11. Available at http://www.harvestplus.org (2013). |
| 41 | Ciolek, A. *et al.* Content of selected nutrients in wheat, barley and oat grain from organic and conventional farming. *J*. *Elementol.* **17,** 181-189 (2012). |
| 42 | Gondek, K. Effect of fertilization with farmyard manure, municipal sewage sludge and compost from biodegradable waste on yield and mineral composition of spring wheat grain. *J*. *Elementol.* **17,** 231-245 (2012). |
| 43 | Rachon, L. *et al.* Comparison of the chemical composition of spring durum wheat grain (*Triticum durum*) and common wheat grain (*Triticum aestivum* ssp. *vulgare*). *J*. *Elementol*. **17,** 105-114 (2012). |
| 44 | Morgounov, A. I. *et al*. Historical changes in grain yield and quality of spring wheat varieties cultivated in Siberia from 1900 to 2010. *Can*. *J*. *Plant Sci*. **93,** 425-433 (2013). |
| 45 | Skrbic, B. *et al.* Principal component analysis of trace elements in Serbian wheat. *J*. *Agr*. *Food Chem*. **53,** 2171-2175 (2005). |
| 46 | Stefanovic, V. Z. *et al*. Undesirable metals content in wheat of different wheat varieties. APTEFF **39,** 69-76 (2008). |
| 47 | Pleijel, H. ＆ Danielsson, H. Yield dilution of grain Zn in wheat grown in open-top chamber experiments with elevated CO_2_ and O_3_ exposure. *J Cereal Sci.* **50,** 278-282 (2009). |
| 48 | Pleijel, H. Effects of ozone on zinc and cadmium accumulation in wheat- dose-response functions and relationship with protein, grain yield, and harvest index. *Ecol. Evol.* **2,** 3186-3194 (2012). |
| 49 | Kirchmann, H. *et al.* Trace element concentration in wheat grain: results from the Swedish long-term soil fertility experiments and national monitoring program. *Environ. Geochem. Hlt.* **31,** 561-571 (2009). |
| 50 | Cooper, J. *et al.* Effect of organic and conventional crop rotation, fertilization, and crop protection practices on metal contents in wheat (*triticum aestivum*). *J. Agr. Food Chem.* **59,** 4715-4724 (2011). |
| 51 | Gooding, M. J. *et al.* Contrasting effects of dwarfing alleles and nitrogen availability on mineral concentrations in wheat grain. *Plant Soil* **360,** 93-107 (2012). |
| 52 | McGrath, S. P. *et al.* Biofortification of zinc in wheat grain by the application of sewage sludge. *Plant Soil* **361,** 97-108 (2012). |
| 53 | EL-Bendary, A. A. *et al.* Zinc efficiency of some Egyptian wheat genotypes grown in Zn-deficient soil. *Int. J. Agri. Science* **3,** 267-274 (2013). |
| 54 | Zeidan, M. S. *et al.* Effect of foliar fertilization of Fe, Mn and Zn on wheat yield and quality in low sandy soils fertility. *World J. Agric. Sci.* **6,** 696-699 (2010). |
| 55 | Abd EI-Ghany, H. M. *et al.* Effect of certain macronutrients foliar application on growth, yield and nutrients content of grains for two bread wheat varieties in sandy soil. *J. Appl. Sci. Res.* **9,** 1110-1115 (2013). |
| 56 | Soleimani, R. Cumulative and residual effects of zinc sulfate on grain yield, zinc, iron, and copper concentration in corn and wheat. *J. Plant Nutr.* **35,** 85-92 (2012). |
| 57 | Bassiri, A. ＆ Nahapetian, A. Differences in concentrations and interrelationships of phytate, phosphorus, magnesium, calcium, zinc, and iron in wheat varieties grown under dryland and irrigated conditions. *J. Agr. Food Chem.* **25,** 1118-1122 (1977). |
| 58 | Karami, M. *et al.* Grain zinc, iron, and copper concentrations of wheat grown in central Iran and their relationships with soil and climate variables. *J. Agr. Food Chem.* **57,** 10876-10882 (2009). |
| 59 | Khoshgoftarmanesh, A. H. *et al*. Zinc efficiency of wheat cultivars grown on a saline calcareous soil. *J. Plant Nutr.* **27,** 1953-1962 (2004). |
| 60 | Pahlavan-Rad, M. R. ＆ Pessarakli, M. Response of wheat plants to zinc, iron, and manganese applications and uptake and concentration of zinc, iron, and manganese in wheat grains. *Commun. Soil Sci. Plant Anal.* **40,** 1322-1332 (2009). |
| 61 | Khoshgoftarmanesh, A. H. *et al.* Classification of wheat genotypes by yield and densities of grain zinc and iron using cluster analysis. *J. Geochem. Expl.* **121,** 49-54 (2012). |
| 62 | Ghasemi, S. *et al.* The effectiveness of foliar applications of synthesized zinc-amino acid chelates in comparison with zinc sulfate to increase yield and grain nutritional quality of wheat. *Eur. J. Agron.* **45,** 68-74 (2013). |
| 63 | Khoshgoftarmanesh, A. H. *et al.* Effect of tire rubber ash and zinc sulfate on yield and grain zinc and cadmium concentrations of different zinc-deficiency tolerance wheat cultivars under field conditions. *Eur. J. Agro.* **49,** 42-49 (2013). |
| 64 | Ayoubi, S. *et al.* Relationships between grain protein, Zn, Cu, Fe and Mn contents in wheat and soil and topographic attributes. *Arch. Agron. Soil Sci.* **60,** 625-638 (2014). |
| 65 | Cakmak, I. *et al.* Zinc and iron concentrations in seeds of wild, primitive, and modern wheats. *Food Nutr. Bull.* **21,** 401-403 (2000). |
| 66 | Cakmak, I. *et al.* Differential response of rye, triticale, bread and durum wheats to zinc deficiency in calcareous soils. *Plant Soil* **188,** 1-10 (1997). |
| 67 | Cakmak, I. *et al*. Biofortification and localization of zinc in wheat grain. *J. Agr. Food Chem.* **58,** 9092-9102 (2010). |
| 68 | Ekiz, H. *et al*. Effects of zinc fertilization and irrigation on grain yield and zinc concentration of various cereals grown in zinc-deficient calcareous soils. *J. Plant Nutr.* **21,** 2245-2256 (1998). |
| 69 | Kalayci, M. *et al*. Grain yield, zinc efficiency and zinc concentration of wheat cultivars grown in a zinc-deficient calcareous soil in field and greenhouse. *Field Crops Res.* **63,** 87-98 (1999). |
| 70 | Yilmaz, A. *et al*. Effect of different zinc application methods on grain yield and zinc concentration in wheat cultivars grown on zinc-deficient calcareous soils. *J. Plant Nutr.* **20,** 461-471 (1997). |
| 71 | Yilmaz, A. *et al.* Effect of seed zinc content on grain yield and zinc concentration of wheat grown in zinc-deficient calcareous soils. *J. Plant Nutr.* **21,** 2257-2264 (1998). |
| 72 | Harmankaya, M. *et al.* Variation of heavy metal and micro and macro element concentrations of bread and durum wheats and their relationship in grain of Turkish wheat cultivars. *Environ. Monit. Assess.* **184,** 5511-5521 (2012). |
| 73 | Abdel-Aal, E. M. *et al.* Phytochemicals and heavy metals content of hairless canary seed: A variety developed for food use. *Lwt-Food Sci. Technol.* **44,** 904-910 (2011). |
| 74 | Gao, X. *et al.* Grain cadmium and zinc concentrations in wheat as affected by genotypic variation and potassium chloride fertilization. *Field Crops Res.* **122,** 95-103 (2011). |
| 75 | Gawalko, E. J. *et al.* Cadmium, copper, iron, manganese, selenium, and zinc in Canadian spring wheat. *Commun. Soil Sci. Plant Anal.* **33,** 3121-3133 (2002). |
| 76 | Gao, X. *et al.* Grain concentrations of protein, iron and zinc and bread making quality in spring wheat as affected by seeding date and nitrogen fertilizer management. *J. Geochem. Expl.* **121,** 6-44 (2012) . |
| 77 | Soltani, S. *et al.* The effect of preceding crop on wheat grain zinc concentration and its relationship to total amino acids and dissolved organic carbon in rhizosphere soil solution. *Biol. Fert. Soils* **50,** 239-247 (2014). |
| 78 | Velu, G. *et al.* Variation for grain micronutrients concentration in wheat core-collection accessions of diverse origin. *Asian J. Crop Sci.* **3,** 43-48 (2011). |
| 79 | Graham, R. *et al*. Breeding for micronutrient density in edible portions of staple food crops: Conventional approaches. *Field Crops Res.* **60,** 57-80 (1999) . |
| 80 | Velu, G. *et al*. Performance of biofortified spring wheat genotypes in target environments for grain zinc and iron concentrations. *Field Crops Res.* **137,** 261-267 (2012). |
| 81 | Moraghan, T. *et al*. Zinc in wheat grain as affected by nitrogen fertilization and available soil zinc. J*.* Plant Nutr*.* **22,** 709-716 (1999). |
| 82 | Murphy, K. M. *et al*. Relationship between yield and mineral nutrient concentrations in historical and modern spring wheat cultivars. *Euphytica* **163,** 381-390 (2008). |
| 83 | Murphy, K. M. *et al*. Nutritional and quality characteristics expressed in 31 perennial wheat breeding lines. *Renew Agr. Food Syst.* **24,** 285-292 (2009). |
| 84 | Pomeranz, Y. ＆ Dikeman, E. Minerals and protein contents in hard red winter wheat flours. *Cereal Chem.* **60,** 80-82 (1983). |
| 85 | Peterson, C. J. *et al.* Influence of cultivar and environment on mineral and protein concentrations of wheat flour, bran and grain. *Cereal Chem.* **63,** 183-186 (1986). |
| 86 | Garvin, D. F. *et al.* Historical shifts in the seed mineral micronutrient concentration of US hard red winter wheat germplasm. *J. Sci. Food Agr.* **86,** 2213-2220 (2006). |
| 87 | Nan, Z. *et al.* Cadmium and zinc interactions and their transfer in soil-crop system under actual field conditions. *Sci. Total Environ.* **285,** 187-195. (2002) |
| 88 | Lavado, R. S. *et al.* Nutrient and heavy metal concentration and distribution in corn, soybean and wheat as affected by different tillage systems in the Argentine Pampas. *Soil Tillage Res.* **62,** 55-60. (2001) |
| 89 | Lavado, R. S. *et al*. Transfer of potentially toxic elements from biosolid-treated soils to maize and wheat crops. *Agr. Ecosyst. Enviro.* **118,** 312-318 (2007). |
| 90 | Podio, N. S. *et al*. Elemental and isotopic fingerprint of argentinean wheat. Matching soil, water, and crop composition to differentiate provenance. *J. Agri. Food Chem.* **61,** 3763-3773 (2013). |
| 91 | Bermudez, G. A. *et al*. Heavy metal and trace element concentrations in wheat grains: Assessment of potential non-carcinogenic health hazard through their consumption. *J. Hazard. Mater.* **193,** 264-271 (2011). |
| 92 | Caires, E. F. *et al*. Surface liming and zinc availability in a long-term experiment under no-till system. *Commun. Soil Sci. Plant Anal.* **40,** 2898-2911 (2009). |
| 93 | Souza, G. A. *et al*. Genotypic variation of zinc and selenium concentration in grains of Brazilian wheat lines. *Plant Sci.* **224**, 27-35 (2014). |
| 94 | Morgounov, A. *et al*. Iron and zinc grain density in common wheat grown in Central Asia. *Euphytica* **155,** 193-203 (2007). |
| 95 | Neelam, K. *et al*. Evaluation and identification of wheat-aegilops addition lines controlling high grain iron and zinc concentration and mugineic acid production. *Cereal Res. Commun.* **40,** 53-61 (2012). |
| 96 | Shivay, Y. S. *et al*. Studies on some nutritional quality parameters of organically or conventionally grown wheat. *Cereal Res. Commun.* **38,** 345-352 (2010). |
| 97 | Shivay, Y. S. *et al*. Effect of zinc-enriched urea on productivity, zinc uptake and efficiency of an aromatic rice-wheat cropping system. *Nutr. Cycl. Agroecosyst.* **81,** 229-243 (2008). |
| 98 | Singh, A. *et al*. Relative effectiveness of various types and methods of zinc application on rice and maize crops grown in calcareous soil. *Plant Soil* **73**, 315-322 (1983). |
| 99 | Srivastava, P. C. *et al*. Comparative study of a Zn-enriched post-methanation bio-sludge and Zn sulfate as Zn sources for a rice-wheat crop rotation. *Nutr. Cycl. Agroecosyst.* **85,** 195-202 (2009). |
| 100 | Verma, T. S. ＆ Minhas, R. S. Zinc and phosphorus interaction in a wheat-maize cropping system. *Fert. Res.* **13,** 77-86 (1987). |
| 101 | Rana, A. *et al*. Biofortification of wheat through inoculation of plant growth promoting rhizobacteria and cyanobacteria. *Eur. J. Soil Biol.* **50,** 118-126 (2012). |
| 102 | Karak, T. ＆ Bhattacharyya, P. Heavy metal accumulation in soil amended with roadside pond sediment and uptake by winter wheat (*Triticum aestivum* L. cv. PBW 343). *Scientific World J.* **10,** 2314-2329 (2010). |
| 103 | Srivastava, P. C. *et al*. Effect of zinc application methods on apparent utilization efficiency of zinc and phosphorus fertilizers under basmati rice-wheat rotation. *Arch. Agron. Soil Sci.* **60,** 33-48 (2014). |
| 104 | Mohan, D. *et al*. Characterization of popular bread wheat cultivars of India for grain quality and the stable genetic resource. *Ind. J. Genet. Plant Breed.* **73,** 14-22 (2013). |
| 105 | Hussain, S. *et al*. Mineral bioavailability in grains of Pakistani bread wheat declines from old to current cultivars. *Euphytica* **186,** 153-163 (2011). |
| 106 | Harris, D. *et al*.'On-farm' seed priming with zinc in chickpea and wheat in Pakistan. *Plant Soil* **306,** 3-10 (2008). |
| 107 | Hussain, S. *et al*. Bioavailable zinc in grains of bread wheat varieties of Pakistan. *Cereal Res. Commun.* **40,** 62-73 (2012). |
| 108 | Hussain, A. *et al*. Concentration of some heavy metals in organically grown primitive, old and modern wheat genotypes**,** Implications for human health. *J. Environ. Sci. Hlth. Par. B. Pest. Food Contam. Agric. Wastes* **47,** 751-758 (2012). |
| 109 | Rafique, E. *et al*. Value of soil zinc balances in predicting fertilizer zinc requirement for cotton-wheat cropping system in irrigated Aridisols. *Plant Soil* **361,**43-55 (2012). |
| 110 | Virk, P. ＆ Velu, G. Zinc wheat. In: conference brief on the 2^nd^ global conference on biofortification: Getting nutritious foods to People. Available at http://biofortconf.ifpri.info (2014). |
| 111 | Guzman, C. *et al*. Use of wheat genetic resources to develop biofortified wheat with enhanced grain zinc and iron concentrations and desirable processing quality. *J. Cereal Sci.* **60,** 617-622 (2014). |
| 112 | Zhao, A.Q. *et al.* Comparison of soil and foliar zinc application for enhancing grain zinc content of wheat when grown on potentially zinc‐deficient calcareous soils. *J. Sci Food Agr.* **94,** 2016-2022 (2014). |
| 113 | Wang, X. *et al.* An effective strategy to improve grain zinc concentration of winter wheat, Aphids prevention and farmers' income. *Field Crop Res.* **184,** 74-79 (2015). |
| 114 | Rakshit, R. *et al.* Super-optimal NPK along with foliar iron application influences bioavailability of iron and zinc of wheat. *Proc. Nat. Acad. Sci. India Sec. B: Biol. Sci.* **86,** 159-164 (2016). |
